# Insights into the Diversity of Secondary Metabolites of *Planktothrix* Using a Biphasic Approach Combining Global Genomics and Metabolomics

**DOI:** 10.3390/toxins11090498

**Published:** 2019-08-27

**Authors:** Sandra Kim Tiam, Muriel Gugger, Justine Demay, Séverine Le Manach, Charlotte Duval, Cécile Bernard, Benjamin Marie

**Affiliations:** 1 Muséum National d’Histoire Naturelle, UMR 7245, CNRS, MNHN Molécules de Communication et Adaptation des Micro-organismes (MCAM), équipe “Cyanobactéries, Cyanotoxines et Environnement”, 12 rue Buffon - RDC bâtiment de cryptogamie - CP 39, 75231 Paris Cedex 05, France; 2 Institut Pasteur, Collection des Cyanobactéries, 28 rue du Dr Roux, 75724 Paris Cedex 05, France

**Keywords:** cyanobacteria, secondary metabolite, genome mining, molecular networking

## Abstract

Cyanobacteria are an ancient lineage of slow-growing photosynthetic bacteria and a prolific source of natural products with diverse chemical structures and potent biological activities and toxicities. The chemical identification of these compounds remains a major bottleneck. Strategies that can prioritize the most prolific strains and novel compounds are of great interest. Here, we combine chemical analysis and genomics to investigate the chemodiversity of secondary metabolites based on their pattern of distribution within some cyanobacteria. *Planktothrix* being a cyanobacterial genus known to form blooms worldwide and to produce a broad spectrum of toxins and other bioactive compounds, we applied this combined approach on four closely related strains of *Planktothrix*. The chemical diversity of the metabolites produced by the four strains was evaluated using an untargeted metabolomics strategy with high-resolution LC–MS. Metabolite profiles were correlated with the potential of metabolite production identified by genomics for the different strains. Although, the *Planktothrix* strains present a global similarity in terms of a biosynthetic cluster gene for microcystin, aeruginosin, and prenylagaramide for example, we found remarkable strain-specific chemodiversity. Only few of the chemical features were common to the four studied strains. Additionally, the MS/MS data were analyzed using Global Natural Products Social Molecular Networking (GNPS) to identify molecular families of the same biosynthetic origin. In conclusion, we depict an efficient, integrative strategy for elucidating the chemical diversity of a given genus and link the data obtained from analytical chemistry to biosynthetic genes of cyanobacteria.

## 1. Introduction

Cyanobacteria are an ancient lineage of bacteria that colonize a broad range of habitats, from soil to oceans, and play important roles in the global nitrogen and carbon cycles [1]. They are also well known for the production of toxins, and to form toxic blooms in freshwater bodies around the world, posing a threat to human health [2,3,4]. However, it has become clear that marine, terrestrial, and freshwater cyanobacteria can produce a large set of natural products in addition to toxic compounds, many of which exhibit remarkable biological activities potentially involved in various ecological or physiological processes [5,6].

The known chemical diversity of cyanobacterial natural products includes over 1100 secondary metabolites [7]. Isolated by means of traditional bioactivity-guided screening techniques [8], these compounds also present promising therapeutic potential, with anticancer, multidrug-reversing, antifungal, antibacterial, anti-inflammatory, antiviral, and potent enzyme-inhibiting bioactivities [9,10]. These molecules can be respectively assigned to a diverse panel of structural classes, including peptides, polyketides, alkaloids, lipids, and terpenes [10,11]. These complex metabolites are commonly synthesized on enzymatic assembly lines of non-ribosomal and ribosomal biosynthetic pathways namely the non-ribosomal peptide synthetase (NRPS) and the polyketide synthase (PKS) enzyme systems [12], or the ribosomally synthesized and post-translationally modified peptide (RiPP) pathways [13].

Genome-mining studies based upon the recent increase of genome sequences available in public databases have demonstrated unexpected diversity and greatly expanded the known distribution of these biosynthetic gene clusters across cyanobacteria [14]. Indeed, genome mining demonstrates that these biosynthetic gene clusters typically encode a large range of auxiliary enzymes that tailor the structure of the secondary metabolites and greatly increase the chemical diversity of the products [15,16]. Hence, the huge variety in genetic organization and various associated tailoring enzymes signify that the currently established chemical diversity remains an underestimation of the achievable potential of these pathways.

Improvements of predictive bioinformatic tools combining established knowledge of secondary metabolism and hidden Markov model–based algorithms such as ClustScan [17], ClusterFinder [18], or antiSMASH [19] have resulted in a more facile identification of core and tailoring enzymes of these pathways, as well as other secondary metabolite gene clusters. These approaches also give us the opportunity to detected gene clusters corresponding to orphans of known products. It now opens new windows for the description of biosynthetic pathways and their respective natural products.

A great perspective for compound identification is expected by combining genome mining with high-throughput global analytical methods. Metabolomics, which aims at providing a holistic investigation of the chemical diversity, brings new highlights on natural-product research. The main bottleneck of this approach remains in the unambiguous identification of the detected compounds. Based on approaches combining high-resolution mass spectrometry (HRMS) equipped with a quadrupole mass filter and coupled to ultra-high-performance liquid chromatography (UHPLC), up-to-date metabolite profiling platforms can rapidly generate accurate structural information for hundreds of metabolites in crude natural extracts. In addition, the development of the Global Natural Products Social Molecular Networking approaches (such as GNPS) has brought opportunities to integrate MS/MS data providing a very powerful tool for molecular annotation [20]. This process compares individual MS/MS fragmentation patterns of each analyte, then constitutes molecular families of structural similarity features, potentially sharing the same biosynthetic origin. This enables the comparison of a high number of samples at once aiding dereplication and tentative structural characterization and constitutes a promising tool especially when integrated with genome information [21,22].

Here, we investigated the metabolite chemodiversity of four monoclonal strains of *Planktothrix,* prominent freshwater bloom-forming cyanobacteria in lakes and reservoirs producing a large set of various bioactive metabolites [23]. To be the most exhaustive and accurate as possible, we performed an integrated global metabolomic and genomic approach, taking advantage of gene and chemical feature analyses with publicly available databases and prediction tools. This approach leads to the description of a large majority of the main secondary metabolites. Their annotations have been possible according to the propagation principle through the clusters, especially thanks to purified standard molecules analyzed under identical experimental conditions.

## 2. Results and Discussion

### 2.1. Biosynthetic Gene Cluster Approach Based on Genome Analysis

The four strains of *Planktothrix* were selected on their green (Pasteur Culture of Cyanobacteria (PCC) 10110, PCC 7805, and NIVA CYA 126/8) vs. red (PCC 7821) morphotypes and their characteristics to produce (PCC 10110, PCC 7821, and NIVA CYA 126/8) or not (PCC 7805) microcystins (Table S1). The on-going genome of PCC 10110 compared to the ones of PCC 7805, PCC 7821, and NIVA CYA 126/8 [24,25] shared a synteny value above 80%, testifying to a global genome conservation between those strains. The clustering of the four strains revealed a close relationship between the two strains PCC 7805 and NIVA 126/8, while the PCC 7821 and the more recently isolated strain from France, PCC 10110, were more distantly related. No valuable distinction between the red/green (i.e., *Planktothrix rubescens*/*agardhii*) morphotypes was observed (Figure S1), but the clustering confirms the separation of the planktonic from the benthic *Planktothrix* eco-species as previously observed [25]. Overall, the genomic comparison between these four strains highlights their global gene content similarities (Figure S2).

The four *Planktothrix* genomes contain distinct backgrounds in terms of biosynthetic gene clusters (Table 1). Up to 11 gene clusters were detected and represented 1%–2% of the whole genome size. In total, three genes clusters coding for three RiPPs and eight genes cluster coding for NRPS and/or PKS were found in these four genomes (Table 1). Seven of these encode the synthesis of already-described metabolite families, including prenylagaramides (*pag*), microviridins (*mdn*), anabaenopeptins (*apt*), cyanopeptolins (*oci*/*mcn*), aeruginosins (*aer*), microcystins (*mcy*), and microginins (*mic*). The shared gene clusters of these known compounds were highly conserved (between 92.0% and 99.4% of amino-acid sequences identity—Table 1). In all four strains, the *apt, mic*, and *oci* genes appeared to be co-localized in a ~70 kb genomic island, as it had been previously showed in *P. rubescens* NIVA CYA 98 [25,26]. In addition to the seven gene clusters corresponding to already-described metabolite families (i.e., *pag*, *mdn*, *apt*, *oci*, *aer*, *mcy,* and *mic*). The gene clusters for *apt*, *mdn*, *mcn*/*oci* were forming similar genomic islands in the strain PCC 10110 than in the three other strains, as recently described [25]. Four other gene clusters encode enzymes for so-far unidentified products (arbitrarily called R1, PNL1, PNL2, and PNL3). Their respective sequences, although presenting the signature of RiPP, NRPS, and/or PKS genes identified by antiSMASH [27], do not exhibit significant similarity in the database. For example, the PNL1 cluster present in PCC 7805 and NIVA CYA 126/8 strains (Figure S3) is tentatively annotated as producing ladderane according to the antiSMASH search. However, these types of compounds are found so far in the membranes of the anammox bacteria, to which the photosynthetic cyanobacteria do not belong.

Overall, this suggests that several common secondary metabolites could be produced by the four strains (six out of the eleven gene clusters were found in more than one strain). In addition, some secondary metabolites will be strain specific as the five remaining clusters were found in only one strain. Note that the partial *apt* sequences of the PCC 7805 strain appear significantly truncated and are very likely not functional. Interestingly, the *aer* cluster of the PCC 7805 strain contains a sequence encoding for a halogenase, suggesting the production of halogenated aeruginosin variants.

The genus *Planktothrix* had been already known for the production of bioactive peptides, including the protein phosphatase inhibiting microcystins, the protease inhibiting cyanopeptolins, aeruginosins, and microviridins, among others [19,28]. A better understanding of the production of these cyanobacterial secondary metabolites has been progressively supported by the elucidation of the synthesis pathways for all the main peptide families: Microcystins [29], aeruginosins [30], anabaenopeptins [31], cyanopeptolins [15], microviridins [32], and prenylagaramides [16]. Individual cyanobacterial clones generally produce a limited number of peptides families. The capacity for the production of these peptide families depends on the presence/absence of the corresponding gene clusters [7,28].

In the last decade, studies have been focused on single-peptide families [30] and, since more recently, global approaches have been undertaken in order to characterize in an exhaustive way the secondary metabolite production potential for a given cyanobacteria strain. Indeed, in the last years, various cyanobacterial genome projects have been initiated in order to detect the occurrence of metabolite biosynthetic pathways. Such recent genomic analyses based on the identification of biosynthetic gene clusters using predictive software are now revealing the extent of the genetic diversity for natural product potential from cyanobacteria, such as *Planktothrix*.

### 2.2. Insights of the Molecular Networking for the Characterization the Chemical Diversity of the Planktothrix Secondary Metabolite Variants

In order to identify *Planktothrix* secondary metabolites, we performed a molecular network based on the global fragmentation pattern profile generated by high-resolution tandem mass spectrometry after UHPLC separation of metabolites in the four strains (Figure S4). Molecular networking utilizes MS/MS data to sort parent ions based on their structural similarity according to their respective fragmentation profiles. Indeed, the secondary ion mass fragmentation data relates directly to molecular structure because chemical bonds break on the basis of bond strength, that relies on respective residue or molecular skeleton structures. The spectral networking of the GNPS algorithm uses the normalized intensity of all fragment ions as independent axes in order to construct multidimensional vectors specific of each spectrum, and to finally compare their similarities using a cosine function and to visualize these relationships between different parent ion masses on a plot diagram representation [33,34].

**Figure 1:**
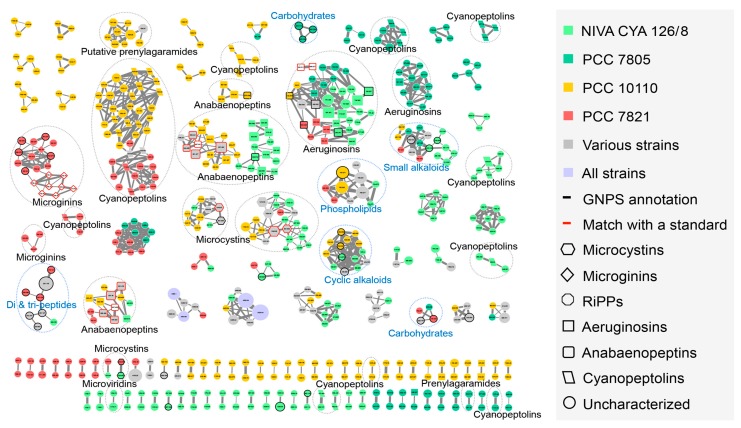
Molecular network generated from MS/MS spectra of the four *Planktothrix* strains using Global Natural Products Social Molecular Networking (GNPS) tool (all data and results are freely available on the GNPS server at the address http://gnps.ucsd.edu/ProteoSAFe/status.jsp?task=98e54f0fa2a84efeb82efa0d24e4d974). The GNPS algorithm compares all MS/MS spectra by aligning them one by one, grouping identical molecules (presenting identical mass and fragmentation pattern) and assigning a cosine score ranking from 0 to 1 to each alignment, allowing network reconstruction of the link between each molecule according to the cosine score calculated between all molecules (cosine score significance threshold set to 0.6). Analytes whose individual masses match with known secondary metabolites from cyanobacteria are indicated with specific shapes. Correspondences with standard molecules from cyanobacteria similarly analyzed or with components from the fragmentation pattern library available from the GNPS server are indicated by heavy red and black lines, respectively. Only clusters of at least two nodes are represented.

The resulting network of the four *Planktothrix* metabolomes was obtained from 2360 analytes and 30 reference compounds. It contains 49 clusters of three or more analytes, regrouping 405 analytes and 69 clusters of two analytes (Figure 1), whereas 429 analytes remain as singles. The molecular clusters were further annotated using the reference compounds and matches with components from libraries publicly available on GNPS platform, such as MassBank, GNPS/NIST14, or EMBL. The nodes grouped in the same molecular clusters (clouds of nodes, linked together according to cosine score >0.6) exhibit similar fragmentation patterns and are specific to the structure of single chemical families. We were thus able to annotate larger clusters according to the fragmentation pattern similarity of their analytes. Some clusters are constituted by ions of primary metabolites such as two clusters of carbohydrates and several single clusters representing for cyclic alkaloids, small alkaloids, phospholipids, and di- and/or tripeptides. In addition, several clusters were containing known secondary metabolites such as seven clusters for cyanopeptolins, three clusters for microcystins, two clusters representing anabaenopeptins, aeruginosins, prenylagaramides, and microginins, together with one for microviridins (Figure 1). The occurrences of these metabolites in the four *Planktothrix* strains are almost perfectly matching with the prediction of presence/absence of their respective gene clusters from genomes (Table 1). This result shows that the large majority of secondary metabolites predicted based on the presence of gene clusters were detected by the LC–MS/MS-based pipeline (Table 2).

In contrast, prenylagaramides were only detected in PCC 7805, PCC 7821, and PCC 10110 and microviridins in NIVA CYA 126/8 (Table 2), while the genome analysis predicted a potential synthesis of both families (both being cyclic peptides produced by RiPP pathways) for all the four studied strains (Table 1). The absence of detection of these compounds during the genome analysis indicated a potential production may have different explanations. First of all, it is possible that these molecules could be produced in minute amount in some strains and thus remained not detectable by our MS/MS-based pipeline based on an intensity-dependent selection step for parent selection (selectivity threshold set to >10,000 counts). Alternatively, it is also very likely that the biosynthetic products present only limited similarity with already described compounds, being not highlighted by the present MS/MS-based annotation process. For example, only three prenylagaramides (prenylagaramides A, B, and C; [16,35]) were structurally characterized while *Planktothrix* genome investigations have revealed many more precursors potentially encoding other prenylagaramide compounds with very diverse predicted structures [16,25]. Thereby, the identification of such cyclic peptides derived from the RiPP gene cluster machineries using MS/MS-based molecular network presents here the main limitation of the global investigation of the metabolite diversity. Indeed, this approach is established on molecular structural similarity of components, and for RiPP cyclic peptides especially, even a little difference, such as a single mutation (e.g., INDELs) in the sequence encoding for their core peptide is susceptible to induce drastic structural dissimilarities of the resulting peptide, making them not suitable for structural similarity–based clustering. On the other side, a similar mutation on the sequence encoding enzymes of the NRPS/PKS complex may have a less drastic consequence on the molecular structure of its end-product [12,13,15]. One complementary approach to specifically detect in these strains all products from these gene clusters could be to perform specific gene cluster deletion to correlate with the disappearance of the production of specific compounds, but this approach still remains technically challenging with *Planktothrix* [29].

However, mass spectrometry–based molecular networking represents a clearly valuable tool for the description of the whole molecular diversity of the cyanobacterial metabolites produced by different strains [36,37,38]. It takes full advantage of the capabilities of modern mass spectrometer–based analytical solutions including the high sensitivity, the high resolution of the molecular mass, the accuracy of the isotopic pattern, the chromatographic retention time, and the fragmentation pattern, together with the use of reference compound libraries for automatic structural identity or analogy search [20,39,40]. Although, no ionization technique is universal, the electrospray ionization (ESI) in the positive mode appears to effectively ionize a wide range of structural classes previously extracted with 80% methanol. According to the present *Planktothrix* strain investigation, this appears to provide a remarkable coverage of these global cyanobacterial metabolomes, as already demonstrated by the *Microcystis* bloom investigation [40].

Cyanobacteria, such as *Planktothrix*, typically produce multiple variants of the same metabolite family [19,41,42]. Much of the metabolite chemical variations can be attributed to a lack of specificity of the NRPS biosynthetic machinery. The production of different variants may indeed be attributed to gene mutations (as for example the gain and loss of genes coding for tailoring enzymes or a modification of the active site inducing a modification of the enzyme substrate affinity) or to change in the availability of the substrates within the cell [43]. It has been proposed that the synthesis of multiple variants of a metabolite family could allow a larger bioactivity panel and confer some plasticity and adaptive advantages for the producing organism [44]. For example, within monospecific blooms of *Planktothrix*, environmental factors drive the dynamics of microcystin and non-microcystin producing strains [45,46]. However, the evolutive and adaptive outcomes of this structural and functional diversification remains poorly investigated [28,47].

Overall, our results demonstrate the efficiency of the GNPS-based analysis of the global chemical diversity of *Planktothrix* metabolites, that remarkably represent a large set of cyclic or non-cyclic peptides biosynthesized by RIPP or PKS/NRPS pathways. In the following sections, we choose to mainly focus our attention on the case of the variant diversity observed within the main microcystin, anabaenopeptin, and aeruginosin clusters.

#### 2.2.1. Microcystins

**Figure 2:**
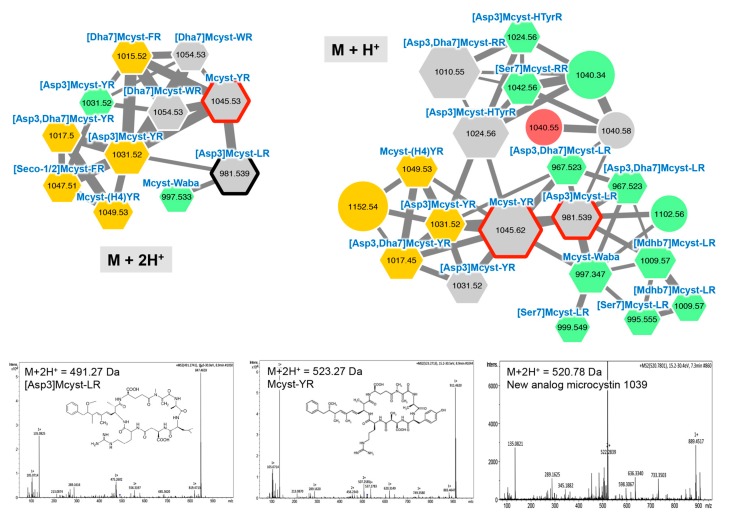
Two main microcystin clusters highlighted by the GNPS analysis based on the MS/MS CID (collision ion dissociation) fragmentation spectra obtained from the *Planktothrix* strains. Analytes whose individual masses match with known microcystins are indicated by hexagons. Correspondences with standard microcystins similarly analyzed or with components, whose fragmentation patterns are available in library of the GNPS server, are indicated by heavy red and black lines, respectively. The color code is similar to the one in the Figure 1 caption. Examples of MS/MS spectra and chemical structures are shown for [Asp3] Mcyst-LR, Mcyst-YR, and a potential new microcystin variant exhibiting a *m*/*z* of 1040.58 Da. Note that (M + H)^+^ and (M + 2H)^2+^ ions are grouped in two distinct molecular clusters.

Some of the cyanobacterial metabolites, such as microcystins, are subject to intensive monitoring due to their role in animal toxicoses [3]. Microcystins are cyclic hepta-peptides that have been firstly described from *Microcystis* and *Planktothrix* [19,45,48]. They are characterized by the presence of a non-proteinaceous amino acid in position 5 (Adda), two amino acids derived from Asp and Glu in position 3 and 6, respectively, and two very variable positions (2 and 4), that serve as reference to name the variant. More than 240 variants have been described so far [49], 215 being references in the microcystin database Toxinmasslist_com_v15b [50]. Two main microcystin molecular clusters were highlighted in the GNPS network. Their identification as microcystin variants was confirmed by the direct matching of two of them according to microcystin standards ([Asp3]-Mcyst-LR and Mcyst-YR) (Figure 2). In these two microcystin clusters, other various ions presented a match between their respective masses and those of microcystin variants previously described [50], while the others, not presenting any match with known microcystin variants may correspond to potential new microcystin variants (e.g., the *m*/*z* 1040.58 node). For these potential new microcystin variants (that does not appear to be adduct according to their respective retention time and mass differences), the observation of their MS/MS spectra shows that they present fragmentation patterns similar to those of other known microcystins (Figure 2). Globally, the three microcystin-producing strains studied here (*Planktothrix* strains PCC 10110, PCC 7821, and NIVA CYA 126/8) exhibit only few common variants (in grey), comprising principally MC-YR, [Asp3]-Mcyst-YR, [Asp3]-Mcyst-LR, among others.

#### 2.2.2. Anabaenopeptins

**Figure 3:**
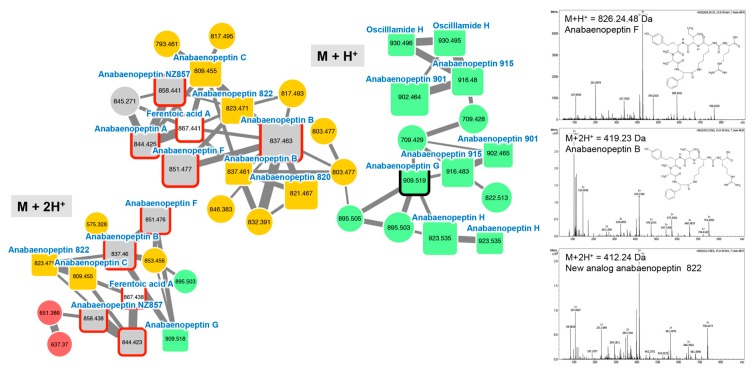
Two anabaenopeptin clusters highlighted by the GNPS analysis based on the MS/MS CID fragmentation spectra obtained from the four *Planktothrix* strains. Analytes whose individual masses match with known anabaenopeptins are indicated by squares with rounded corners. Correspondences with standard anabaenopeptins similarly analyzed or with components, whose fragmentation patterns are available in the library of the GNPS server, are indicated by heavy red and black lines, respectively. The color code is similar to the one in the Figure 1 caption. Example of MS/MS spectra and chemical structures are shown for anabaenopeptin F, anabaenopeptin B, and a potential new anabaenopeptin 822 variant exhibiting a *m*/*z* of 823.47 Da. Note that (M + H)^+^ and (M + 2H)^2+^ ions are grouped in two distinct clusters.

Anabaenopeptins constitute a very diverse family of cyclic hexa-peptides described from *Microcystis*, *Planktothrix*, *Anabaena*, *Aphanizomenon*, and *Nostoc* (see review in [51]). Up to 75 anabaenopeptin variants have been described so far [41]. Except for the D-Lys (position 2) that is linked to the carboxylic group of the amino acid placed in position 6, all other positions are variable allowing a large structural diversity of the family whose molecules exhibit masses between 750 and 950 Da [52]. Two main anabaenopeptin clusters (M + H^+^ and M + 2H^2+^ clusters) were highlighted in the GNPS network according to the formal identification of six standard molecules (anabaenopeptin A, B, F, G, NZ857, and ferentoic acid A) analyzed in parallel for the four *Planktothrix* extracts with the same protocol (Figure 3). Other components of these molecular clusters correspond to ions presenting a match with the mass of other anabaenopeptin variants previously described [41], or for more than one-third of them to compounds that very likely correspond to potentially new anabaenopeptin variants.

#### 2.2.3. Aeruginosins and Halogenation

**Figure 4:**
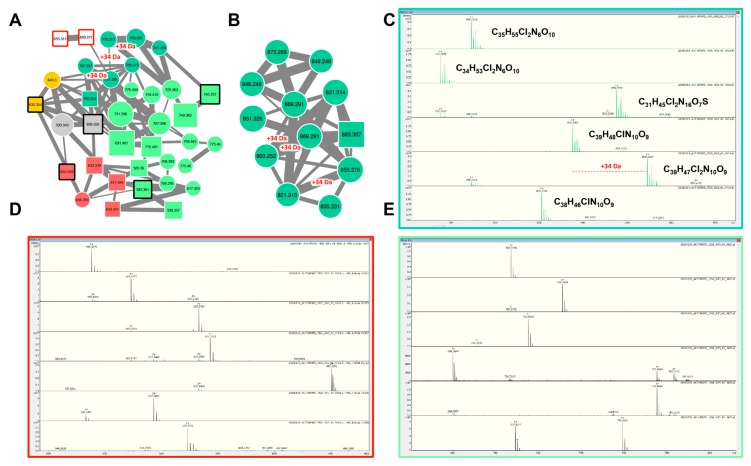
Two aeruginosin clusters highlighted by the GNPS analysis based on the MS/MS CID fragmentation spectra obtained from the four *Planktothrix* strains. Analytes whose individual masses match with known aeruginosins are indicated as squares with sharp corners (**A**,**B**). Correspondences with standard aeruginosins similarly analyzed or with components, whose fragmentation patterns are available in library of the GNPS server, are indicated by heavy red and black lines, respectively. The color code is similar to the one in Figure 1. Selection of isotopic pattern of various aeruginosins observed in the strains PCC 7805 (**C**), PCC 7821 (**D**), and PCC 10110 (**E**), respectively. Note that these isotopic patterns indicate the presence of mono- or di-chlorinations exclusively for aeruginosins of the PCC 7805 strain. The +34 Da mass shift between potentially non-, mono-, and di-chlorinated molecules is indicated in red.

Aeruginosins constitute a linear tetra-peptide family of more than 94 variants described so far [41]. Their MS/MS fragmentation patterns are often characterized by the presence of a Choi fragment (immonium with 140.109 *m*/*z*) and other recurrent fragments from Hpla or Pla residues [19]. Their composition is rather variable, and the component of this family exhibit masses comprised between 430 and 900 Da [52]. The molecular network obtained from the four *Planktothrix* strains exhibits two aeruginosin clusters (Figure 4) that were highlighted by the presence of seven standard molecules. Other components of these molecular clusters correspond to ions presenting a mass match with other variants of aeruginosin previously described [41], and up to 58% of all these compounds may represent potential new aeruginosin variants. Interestingly, we noticed that several aeruginosin variants of the PCC 7805 (shown in dark green in Figure 4A) show characteristic isotopic pattern of mono- or di-chlorination, as recently illustrated for this strain by Briand and co-workers [53]. These observations are in agreement with genomic analysis that detects a non-heme iron O_2_-dependent halogenase, potentially involved in the halogenation of biosynthetic products in the *aer* cluster of this strain (Table 1).

### 2.3. Global Capability to Annotate Cyanobacterial Metabolites

**Figure 5:**
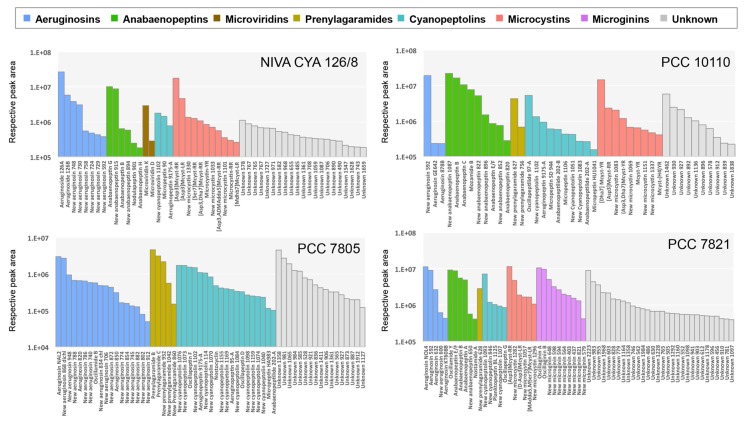
Main secondary metabolites detected by mass spectrometry in the four *Planktothrix* strains and their respective annotations (Supplementary Data S1–S4) according to their direct identification, thanks to analytical standards or their belonging to GNPS molecular clusters (Figure 1).

On the one hand, the potential of secondary metabolite production of the four *Planktothrix* strains was investigated by genome mining approach using RiPP, NRPS, and/or PKS gene cluster search software antiSMASH 4.0 (Table 1). This investigation suggests that various metabolite families (i.e., microcystins, anabaenopeptins, aeruginosins, cyanopeptolins, microviridins, microviridins, and prenylagaramides) could be synthetized by these different strains. On the other hand, the molecular diversity of the produced metabolites was investigated by high-resolution mass spectrometry–based analyses. In this approach, molecules could be identified according to their molecular formula (estimated according to their accurate mass and isotopic pattern), their retention time, and the presence of qualifying ions in their MS/MS fragmentation spectra. Such direct identifications (referred here as “gold” annotations) are supported by a specific database of analytical standard molecules. The whole analysis of MS/MS spectra by GNPS molecular networking gave the opportunity to propose even more annotations for the molecules whose analytical standards are lacking (that is the case of most of the cyanobacterial metabolites), according to a logical annotation-by-propagation principle. Indeed, the analytes that belong to a molecular cluster that present a match with a standard can be annotated by extension, as “silver” annotation, when the molecule presents a mass that corresponds to an already known cyanobacterial metabolite [41] or as “bronze” annotation when no known metabolite corresponds, suggesting this analyte concerns a potentially new variant. Following these different criteria of identification, the 40–60 most intensive ions analyzed by mass spectrometry have been manually annotated (Figure 5 and Supplementary Data S1–S4).

This combined effort performed here on four *Planktothrix* strains illustrates remarkably the efficiency and the congruency of the two-omic approaches, both of which rely highly on automatized pipelines. Taken together, these two complementary investigations lead to the annotation of most of the main metabolites of the four investigated *Planktothrix* strains. Indeed, with a good level of certainty (gold, silver, or bronze annotations), up to 77% of the above 40–60 most intense metabolites could have been annotated, while only less than 10% could be directly annotated thanks to analytical standards only (gold annotation). To our knowledge, this analysis represents one of the most complete investigation of the chemodiversity of metabolites produced by *Planktothrix* strains available so far, in term of the set of both cluster and analyte annotations.

By leading to the global annotation of most of the secondary metabolites (i.e., RiPP, NRPS/PKS), the present work illustrates the strength and the promising perspective that are offered by such joint genomic and metabolomic investigation of the chemical richness supported by clonal cultures of micro-organisms, such as cyanobacteria [54], heterotrophic bacteria [55], or fungi [56].

## 3. Conclusions

While genome mining investigation through biosynthetic gene cluster search provides basic information on the metabolite molecular families that might be produced by an organism [57], the mass spectrometric molecular networking constitutes a remarkable tool for the direct identification of structural analogues within a set of chemical extracts [41]. Combining these innovative global approaches can help with dereplication and to identify interesting targets for chemical isolation. This constitutes powerful and orthogonal means that support the novel natural product discovery and the in-depth strain characterization [21,54,57,58]. Such approaches appear to be very useful in order to shed light on both hot-spot strains presenting specific chemical diversity and specific molecular families that aim at being further structurally and biologically characterized.

We thus recommend a first screening with genome mining in order to select the cyanobacterial strains of interest based on their secondary metabolite production potential. In a second step, the effective production of secondary metabolites can be characterized in order to verify the prediction based on genome mining. For that commercial standards, databases on fragmentation mass spectra, and molecular networking analyses can be used together in order to obtain a precise description of the secondary metabolite diversity.

In the near future, we expect an even deeper global metabolome characterization thanks to chemo- and bio-informatic tools that are currently in development and aim at better predicting the structure of novel analogues by in silico MS/MS fragmentation [59,60], the de novo sequencing of circularized and modified peptides [61], or the structure prediction of biosynthetic products based on cluster gene sequences [27].

## 4. Materials and Methods

### 4.1. Planktothrix Strains and Culture

*P. agardhii* PCC 10110 isolated from a 2001 bloom sample in a Paris suburban area is a green-pigmented strain producing microcystins. *P. agardhii* NIVA-CYA126/8, green pigmented, and *P. rubescens* PCC 7821, red pigmented, were both microcystin producers and originated from Nordic lakes. *P. agardhii* PCC 7805 isolated from a temperate lake in the Netherlands does not produce microcystins. The four strains were grown in BG11 media at 18 °C for PCC 7821, and at 22 °C for the three others. The cultures were maintained in 2 L Erlenmeyer flasks with a photon flux density of 6 µmol·M^−2^S^−1^ and a 13:11 h light:dark cycle. Fresh cultures were inoculated every four weeks to promote optimal growth.

### 4.2. Description of the DNA Isolation, Sequencing, and Assembling Methods

The on-going genome of *P. agardhii* PCC 10110 was obtained from a 40 mL culture. The data of the gene cluster analyzed here are freely available on request to the authors. Nucleic acid extraction of cyanobacterial cells to obtain DNA was carried out as previously described [62]. Genome sequencing was performed by the Mutualized Platform for Microbiology at Institut Pasteur. The whole-genome sequencing was carried out using the Nextera XT DNA sample preparation kit (Illumina) for 2 × 150 bps paired-end reads (insert size ~300 bps). All sequenced paired-end reads were clipped and trimmed with AlienTrimmer*2* (v. 0.4.0), and subjected to a sequencing error correction with Musket*3* (v. 1.1) as well as a digital normalization procedure with khmer*4* (v. 1.3). For each sample, remaining processed reads were assembled with SPAdes*5* (v. 3.7.0). The genome was further integrated in the MicroScope platform v3.12.2 (http://www.genoscope.cns.fr/agc/microscope) similar to the genomes of PCC 7805, PCC 7821, and NIVA CYA 126/8 [24,25,63].

### 4.3. In Silico Analyses in MicroScope

The clustering of the strain PCC 10110 was performed with the three other published genomes to display the genomic similarity between these strains. This clustering was computed from all-pairs distances ≤0.06 (~94% ANI) that correspond to the ANI standard to define a species group. In addition, natural product gene clusters, including NRPS, PKS, and RiPP genes, were identified using the antiSMASH 4.0 software [27] available through MicroScope platform.

### 4.4. Metabolome Biomass Extraction and Analysis by Mass Spectrometry

The biomass (20 mL) of the four *Planktothrix* strains cultured in triplicate was centrifuged at 4000 rpm for 10 min. The supernatants were discarded, the pellets were freeze-dried and lyophilized. Firstly, the metabolite extraction efficiency was tested on the four strains testing four procedures (A—single extraction with ACN/MetOH/H_2_O 50%/30%/20% *v/v*, B—single extraction with MetOH/H_2_O 80%/20% *v/v*, C—single extraction with MetOH/H_2_O 30%/70% *v/v*, and D—double extraction with MetOH/H_2_O 30%/70% *v/v*, then with MetOH/H_2_O 80%/20% *v/v*). The extraction “B” showed the best efficiency with results similar to the ones of the double extraction procedure and, thus, was then further selected. Then, the lyophilized cells were weighted and sonicated for 2 min in 80% methanol with a constant ratio of 100 µL of solvent for 1 mg of dried biomass and centrifuged at 4 °C (12,000× *g*; 5 min). Two microliters of the supernatant representing the metabolite extracts were then analyzed in triplicate on an ultra-high-performance liquid chromatograph (UHPLC Ultimate 3000, Thermo, Waltham, MA, USA) using a Polar Advances II 2.5 pore C_18_ column (Thermo, Waltham, MA, USA) at a 300 µL·min^−1^ flow rate with a linear gradient of acetonitrile in 0.1% formic acid (5%–90% in 21 min) coupled with a high-resolution mass spectrometer. The eluted metabolite contents were analyzed using an electrospray ionization hybrid quadrupole time-of-flight (ESI-QqTOF) high-resolution mass spectrometer (Maxis II ETD, Bruker) on positive simple MS or on positive autoMS/MS mode with information-dependent acquisition (IDA), on the 50–1500 *m*/*z* range at 2 Hz or between 2 and 8 Hz speed, for MS and MS/MS respectively, according to relative intensity of parent ions, in consecutive cycle times of 2.5 s, with an active exclusion of previously analyzed parents. The data were analyzed with the DataAnalysis 4.4 software for internal recalibration (<0.5 ppm) and MGF exports were generated from MS/MS spectra between 1 and 15 min. The raw MS and MS/MS data were investigated with MetaboScape 4.0 software (Bruker, Bremen, Germany) in order to automatically search and group together all classical adduct forms ([M + H]^+^, [M + 2H]^+^, [M + 3H]^+^, [M + Na]^+^, [M + K]^+^, and [M + NH_4_]^+^) using a threshold value of 0.8 for the co-elution coefficient factor. Metabolite annotation was attempted according to the precise mass of the molecules and their respective MS/MS fragmentation patterns with regards to an in-house database of above 700 cyanobacteria metabolites and confirmed with 36 commercially available standard molecules from the various cyanobacterial specific metabolite families (e.g., cyanopeptolins, aeruginosins, microginins, anabaenopeptins, aerucyclamides, microcystins, saxitoxins, anatoxins, and cylindrospermopsins) analyzed similarly in our mass spectrometry platform.

### 4.5. Data Treatment and Molecular Networking

Using the whole MS/MS data (converted in mgf format) obtained for the four strains taken together, a molecular network was created using the online workflow at Global Natural Products Social Molecular Networking (GNPS) (http://gnps.ucsd.edu) [20]. The data were then clustered with MS-Cluster with a parent mass tolerance of 1.0 Da and an MS/MS fragment ion tolerance of 0.5 Da to create consensus spectra. Consensus spectra that contained less than two spectra were discarded. A network was then created where edges were filtered to have a cosine score above 0.6 and more than five matched peaks. Further edges between two nodes were kept in the network only if each of the nodes appeared in each other’s respective top 10 most similar nodes. The spectra in the network were then searched against the GNPS spectral libraries. All matches kept between network spectra and library spectra were required to have a score above 0.6 and at least five matched peaks. All results are freely available on the GNPS server (http://gnps.ucsd.edu/ProteoSAFe/status.jsp?task=98e54f0fa2a84efeb82efa0d24e4d974). The clustered spectra of the network were annotated by comparing monoisotopic mass to our in-house cyanobacteria metabolite databases according to MS and MS/MS fragmentation pattern matches. Molecular networks were visualized using Cytoscape 3.6.0.

## Figures and Tables

**Table 1:** Distribution and diversity of the gene clusters involved in the biosynthesis of natural products of the studied *Planktothrix*. These gene clusters comprise ribosomally synthesized and post-translationally modified peptides (RiPPs), non-ribosomal peptide synthetase (NRPS), and polyketide synthase (PKS). Genetic distances are calculated according to a reference (Ref) and are expressed as percentage of amino acid identity. h: Halogenase-containing cluster; s: Shorter *mcyA* sequence; m: Sequence presents on megaplasmid; * from the same genetic island.

Synthetic Pathways	RiPP			NRPS and/or PKS							
*Planktothrix* Strains/Clusters	Prenylagaramide (*pag*)	Unknown R1	Microviridin (*mdn*) *	Anabaenopeptin (*apt*) *	Cyanopeptolin (*mcn/oci*) *	Aeruginosin (*aer*)	Microcystin (*mcy*)	Microginin (*mic*)	Unknown PNL1	Unknown PNL2	Unknown PNL3
PCC 7821	Ref	–	Ref	Ref	Ref	Ref	Ref ^s^	Ref ^m^	–	Ref	–
PCC 7805	92.1	–	92	Partial	96.6	97.0 ^h^	–	–	Ref ^m^	–	–
PCC 10110	98.3	–	96.4	98.2	98.7	98.3	99	–	–	–	–
NIVA CYA 126/8	91.7	Ref	94.7	92.2	97.1	96.3	99.4	–	97.3	–	Ref

**Table 2:** Distribution and diversity of metabolite produced by the four *Planktothrix* strains according to metabolomic dataset investigated with GNPS (Figure 2). Green “YES” and red “NO” indicate when the gene cluster and the corresponding metabolite are present, or not, respectively. “N.D.” indicates when no metabolite was detected despite the presence of its specific genetic information in the respective genome.

Synthetic Pathways	RiPP		NRPS and/or PKS					Others		
*Planktothrix* Strains/Molecular Family	Prenylagaramide	Microviridin	Anabaenopeptin	Cyanopeptolin	Aeruginosin	Microcystin	Microginin	Phospholipid	Small Alkaloid	Carbohydrate
PCC 7821	YES	**N.D.**	**YES**	**YES**	**YES**	**YES**	**YES**	**YES**	**YES**	**YES**
PCC 7805	**YES**	**N.D.**	**NO**	**YES**	**YES^h^**	**NO**	**NO**	**YES**	**YES**	**YES**
PCC 10110	**YES**	**N.D.**	**YES**	**YES**	**YES**	**YES**	**NO**	**YES**	**YES**	**YES**
NIVA CYA 126/8	**N.D.**	**YES**	**YES**	**YES**	**YES**	**YES**	**NO**	**YES**	**YES**	**N.D.**

^h^ indicates that several of this relative molecules present halogenations.

## Supplementary Materials

The following are available online at https://www.mdpi.com/2072-6651/11/9/498/s1, Table S1: Main characteristics of the four *Planktothrix* strains, Figure S1: Clustering of *Planktothrix* visualized on the MicroScope platform, Figure S2: Venn diagram of the pan-genomes of the four *Planktothrix* strains studied using the MICFAM tool computing the SiLiX software available on MicroScope platform, Figure S3: Cluster PNL1 of unknown products, Figure S4: Quality controls of the MS/MS dataset used for the molecular networking, Data S1–S4: List of annotated metabolites for the NIVA CYA 126/8, PCC 10110, PCC 7805, and PCC 7821 strains, respectively.
