# Supplementary material for: Insights into the Diversity of Secondary Metabolites of Planktothrix Using a Biphasic Approach Combining Global Genomics and Metabolomics
Source: Toxins (Basel). 2019 Aug 27;11(9):498. doi: 10.3390/toxins11090498 (PMC6784222; doi:10.3390/toxins11090498)
Supplement: Supplementary file 1 [file toxins-11-00498-s001.zip › toxins-549555 SI.docx]

Supplementary Materials: Insights into the Diversity of Secondary Metabolites of *Planktothrix* Using a Biphasic Approach Combining Global Genomics and Metabolomics

Sandra Kim Tiam, Muriel Gugger, Justine Demay, Séverine Le Manach, Charlotte Duval, Cécile Bernard and Benjamin Marie

**Table S1.** Main characteristics of the 4 *Planktothrix* strains

| ***Planktothrix* Strains** | **PCC 7805** | **PCC 10110** | **PCC 7821** | **NIVA CYA 126-8** |
| --- | --- | --- | --- | --- |
| Previously named | *P. agardhii* | *P. agardhii* | *P. rubescens* | *P. agardhii* |
| Phenotype (color) | Green | Green | Red | Green |
| Major phycobiliprotein | Phycocyanin | Phycocyanin | Phycoerythrin | Phycocyanin |
| Microcystin synthesis | ❌ | ✔ | ✔ | ✔ |
| Habitat | Planktic | Planktic | Planktic | Planktic |
| Origin | Temperate lake,  Netherlands | Temperate  lake, France | Nordic lake | Nordic lake |


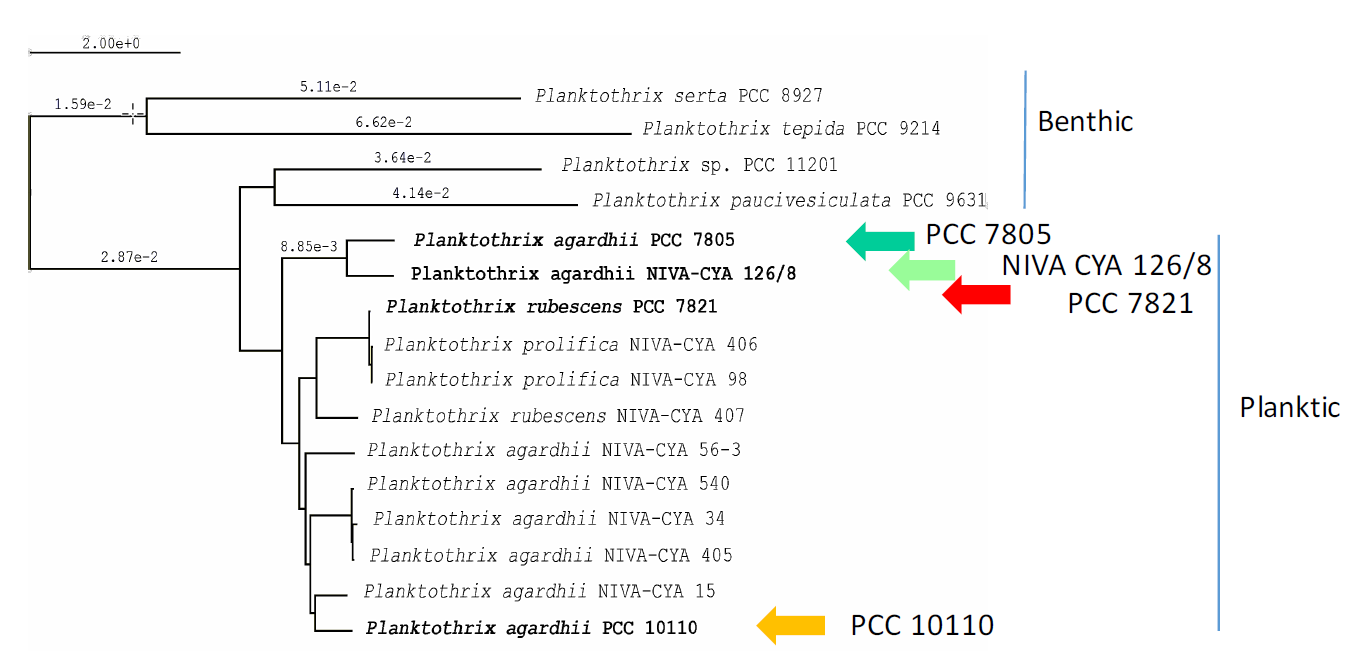


**Figure S1.** Clustering of the of *Planktothrix* visualized on the MicroScope platform (http://www.genoscope.cns.fr/agc/microscope). The genomic similarity is estimated using Mash, a software that computes a distance between two genomes. From all the pairwise distances of the genomes set, a tree is construct dynamically using the neighbor-joining javascript package. The tree displays clustering annotations. This clustering has been computed from all-pairs distances ≤0.06 (≈94% ANI) that correspond to the ANI standard to define a species group.


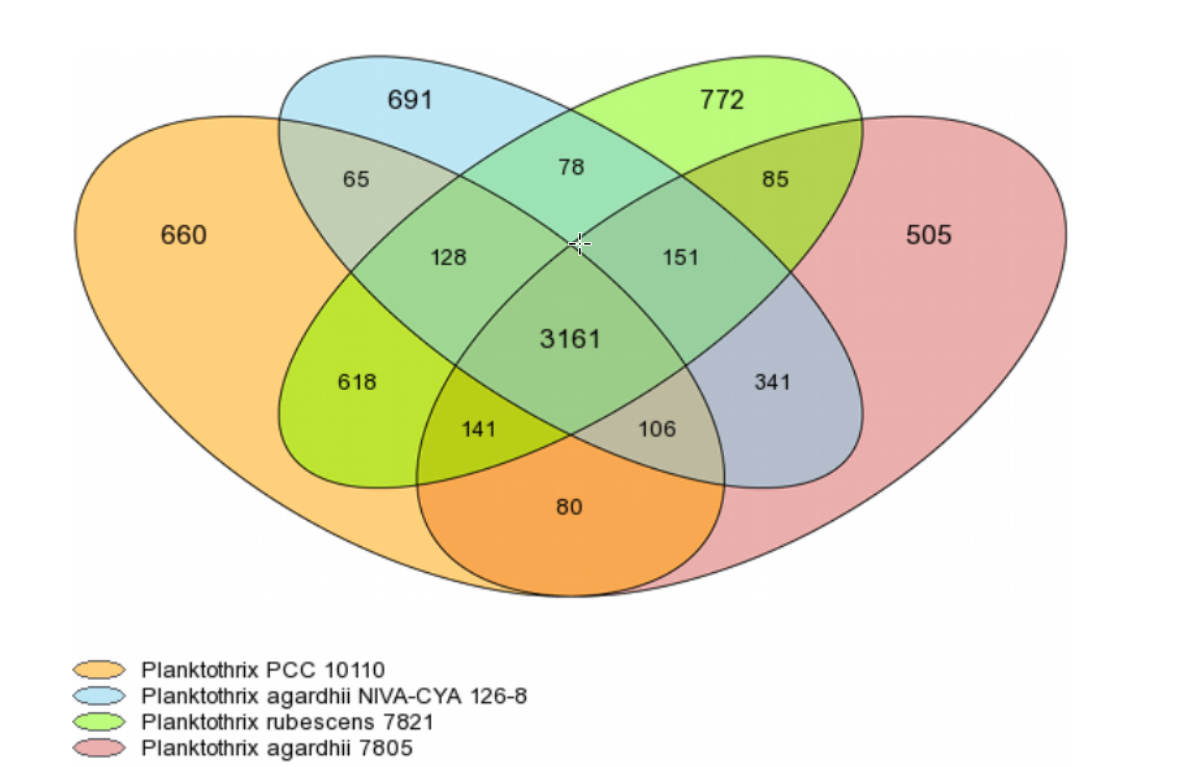


**Supplementary figure S2.** Venn diagram of the pan-genomes (determining the common part = core-genome, or variable portion of the genome = variable-genome) of the 4 *Planktothrix* strains using the MICFAM tool computing the SiLiX software available on MicroScope platform.


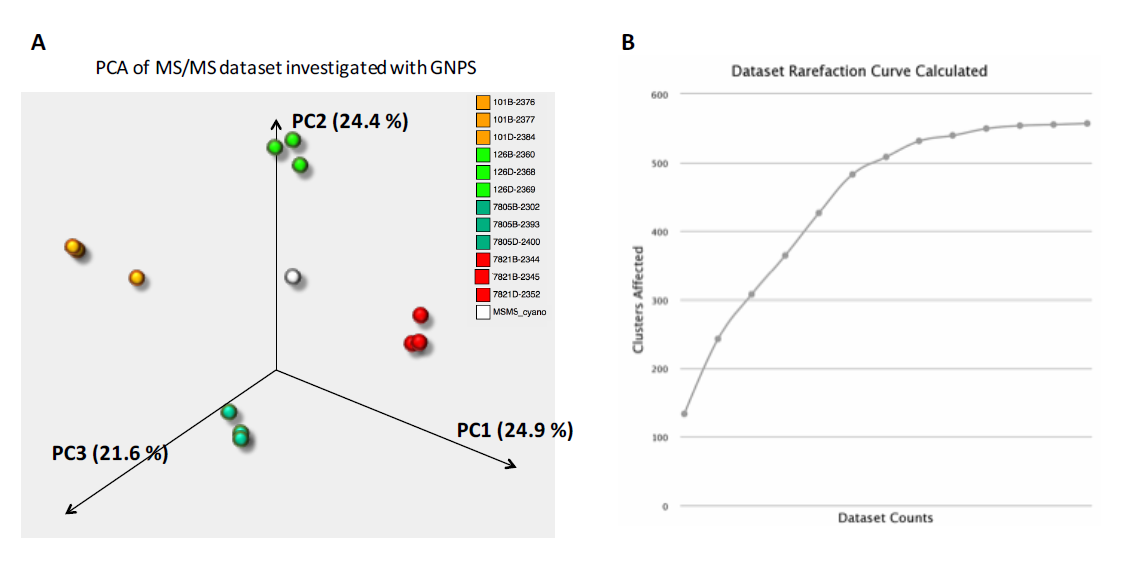


**Figure S3.** (**A**) Principal component analysis (PCA) of the MS/MS data set generated for each one of the four analyzed in triplicat , together with the experimental MS/MS spectra list obtained for 30 purified cyanobacterial metabolites similarly analyzed (MSMS_cyano). (**B**) Rarefaction curve of the MS/MS cluster affection illustrates the presentativeness of the GNPS network for the global description of the 4 strains *Planktothrix* metabolomic dataset.
